# Field Evidence of Social Influence in the Expression of Political Preferences: The Case of Secessionists Flags in Barcelona

**DOI:** 10.1371/journal.pone.0125085

**Published:** 2015-05-11

**Authors:** Antonio Parravano, José A. Noguera, Paula Hermida, Jordi Tena-Sánchez

**Affiliations:** 1 Universidad de Los Andes, Centro de Física Fundamental, Mérida, Venezuela; 2 Instituto de Estudios Sociales Avanzados (IESA-CSIC), Córdoba 14004, Spain; 3 Universitat Autònoma de Barcelona, Group of Analytical Sociology and Institutional Design (GSADI), Barcelona, Spain; University Toulouse 1 Capitole, FRANCE

## Abstract

Models of social influence have explored the dynamics of social contagion, imitation, and diffusion of different types of traits, opinions, and conducts. However, few behavioral data indicating social influence dynamics have been obtained from direct observation in “natural” social contexts. The present research provides that kind of evidence in the case of the public expression of political preferences in the city of Barcelona, where thousands of citizens supporting the secession of Catalonia from Spain have placed a Catalan flag in their balconies and windows. Here we present two different studies. 1) During July 2013 we registered the number of flags in 26% of the electoral districts in the city of Barcelona. We find that there is a large dispersion in the density of flags in districts with similar density of pro-independence voters. However, by comparing the moving average to the global mean we find that the density of flags tends to be fostered in electoral districts where there is a clear majority of pro-independence vote, while it is inhibited in the opposite cases. We also show that the distribution of flags in the observed districts deviates significantly from that of an equivalent random distribution. 2) During 17 days around Catalonia’s 2013 national holiday we observed the position at balcony resolution of the flags displayed in the facades of a sub-sample of 82 blocks. We compare the ‘clustering index’ of flags on the facades observed each day to thousands of equivalent random distributions. Again we provide evidence that successive hangings of flags are not independent events but that a local influence mechanism is favoring their clustering. We also find that except for the national holiday day the density of flags tends to be fostered in facades located in electoral districts where there is a clear majority of pro-independence vote.

## Introduction

People’s decisions, opinions and behavior partially depend on what others decide, think and do [1–3]. The concept of social influence refers to the fact that in many social contexts the probability of an individual acting in a given way depends on how many individuals have already acted in that way [4–6]. Therefore the social diffusion of a given behavior may be typically affected by the perception people has of how the members of the relevant group are behaving. As a mechanism to explain social diffusion, social influence (adjusting to perceived collective behavior) has been distinguished from social contagion (start doing A when you contact someone doing A), from rational imitation (under uncertainty, do as everybody else does) and from social learning (do A when you see A works fine for others) [7, 8]. Different models have explored the dynamics of social influence of many types of social and political behaviors, opinions and traits. The spread of obesity, smoking, alcohol consumption, happiness, divorce, suicide, sexual practices, tastes in music, books and movies, altruism, political mobilization, electoral preferences, and many other conducts, beliefs and preferences has been shown to be afected by social influence [1, 2, 5, 7–21].

Leaving aside purely theoretical models, most empirical studies to date rely on four different sources of evidence: surveys or longitudinal data [14, 22–24], virtual networks and internet behavior [6, 10, 25–28], historical records [29, 30], and experiments [2, 31–35]. In contrast with the vast majority of studies in this field, in this article we present behavioral evidence obtained from direct observation in a particular social context: the public expression of political preferences in the city of Barcelona during July and September 2013, through the placing of Catalan secessionist flags in balconies and windows.

This paper makes several contributions: first, it provides a case-study of a social influence process by direct observation of an objective behavior in a real context; this allows to discard possible biases introduced by subjectivity in self-reporting (as in survey evidence), artificial situations such as experimental treatments or survey interviews, and virtual behavior in the net as opposed to interaction in physical social contexts. Second, our study provides evidence that not only political preferences are affected by social influence [13, 25], but also its public expression; this is important since the literature has widely discussed cases of spiral of silence or pluralistic ignorance regarding political preferences [36–39], but large empirical data on such cases are still scarce. Third, our research also gives empirical support to the thesis that social influence has a strong spatial dimension [14, 17, 40], that is, that the probability of an agent being socially influenced by others is higher the smaller is the physical distance between them.

The observed behavior (hanging a flag in private households’ windows and balconies) is a promising candidate to be affected by social influence: it is easily observable for any individual in the area, it has a simple binary structure (to hang or not to hang a flag), and a very clear meaning (to express support for the Catalan secessionist process). It is a case of influence that results from the need to identify oneself as a member of a group in terms of political preferences, and to signal it to the world [2, 32].

However, some clarifications are due. First, we assume that social influence generated by flag-hanging would not be affecting political preferences as such, but their expression through the successive placing of flags; political support for Catalan independence is most likely to be produced by factors other than seeing flags. What we want to determine is whether an individual already having pro-independence preferences will tend more or less to hang a flag depending on how many others do so. Second, our focus is on whether the observed distribution of flags indicates the existence of social influence, not which specific social influence mechanism is in place; we show that social influence is necessary to explain the observed distribution, but several different mechanisms (or combination of mechanisms) might be consistent with the pattern (this is a usual problem of studies on social influence: [7, 19]). However, some constraints on the spatial scale of the influence mechanisms can be established. Therefore, our approach does not preclude that other possible mechanisms of diffusion such as dyadic social contagion or global common stimuli (such as media effects) may be acting simultaneously to collective social influence.

Since a preliminary observation of the main streets and avenues of the city in January 2013 suggested that flags tend to appear together in clusters, and that there is not a linear relationship between the frequency of flags and variables such as voting behavior or income level, we gather systematic observational data in order to test whether flags are clustered in a non-random way, and whether its distribution is completely explained by voting behavior or a social influence process may also be in place. Our hypothesis is that the probability of placing a Catalan flag in a given household’s balcony or window is correlated with voting for secessionist parties, but also significantly affected by the number of neighbors who hang a flag. We test this hypothesis by analyzing two data sets: one at the meso level where the units of observation are electoral districts, and other at the micro level where the units of observation are household’s windows or balconies. The quantitative characterizations of the state of the system at both the mesoscale and at the microscale, as well as the reaction of the system to the influence of the national holiday, are valuable constraints for theoretical models.

### The case: flag-hanging behavior and the Catalan secessionist process

On Catalonia’s national day (11 September, the ‘Diada’), lots of people traditionally hang a Catalan flag in their balconies or windows, and remove it the day after. But since the 2012 Diada, thousands of flags stay hanged, and others appear. The reason is that many citizens want to express their political support for Catalonia’s secessionist process started that year by the nationalist Catalan government.

From 2005 onwards, the constitutional status of Catalonia within the Spanish state has been subject to strong political struggle and discussion. In 2006, the Spanish Parliament significantly cut Catalan aspirations in the proposal for a new Autonomy Statutory Act, and in 2010 the Spanish Constitutional Court abolished important parts of the Catalan Statutory Act which was approved in referendum in 2006. During all this period, political struggle between Catalan and Spanish governments on funding and linguistic rights has dominated the political agenda. This situation raised massive demonstrations in Barcelona on a yearly basis from 2010, until the Catalan president, echoing a widespread social and political mobilization, proposed to celebrate a referendum on independence from Spain and called for early Catalan elections in 2012. In this election, secessionist parties won a big majority of the Parliament. In 2013 four pro-referendum parties agreed on celebrating the referendum on November 9th 2014. The Spanish government has refused to negotiate. In September 2014, the Catalan government officially called for the referendum, but the Spanish Constitutional Court suspended the call.

In this context and since September 2012, Catalan flags have proliferated in Barcelona’s balconies and windows. The act of hanging a Catalan flag in a flat’s balcony or window has become a usual and visible (though far from majority) way of expressing support for ‘the process’ (a term secessionists use to name the political and social road to independence). There is little doubt that someone placing a Catalan flag in his household’s forefront is clearly expressing support for celebrating a referendum, and most likely also for independence. The difference is relevant in some cases because the agreed question for the proposed referendum includes a third option that Catalonia becomes a non-independent state. Besides, two of the pro-referendum parties (Convergència i Unió, CiU and Iniciativa per Catalunya-Verds, ICV) are not unanimous on their support to independence. However, all polls show that only a very slight fraction of voters (including voters of those two parties) would opt for the ‘third way’ rather than for secession or the status quo. Even if not absolutely all the people who hang a flag might vote for independence, it is quite granted that none of them would vote for the status quo and that they all support the referendum. For simplicity, we will use the term ‘pro-referendum’ instead of ‘pro-independence’ from now on.

## Methods

### First study

**Fig 1:**
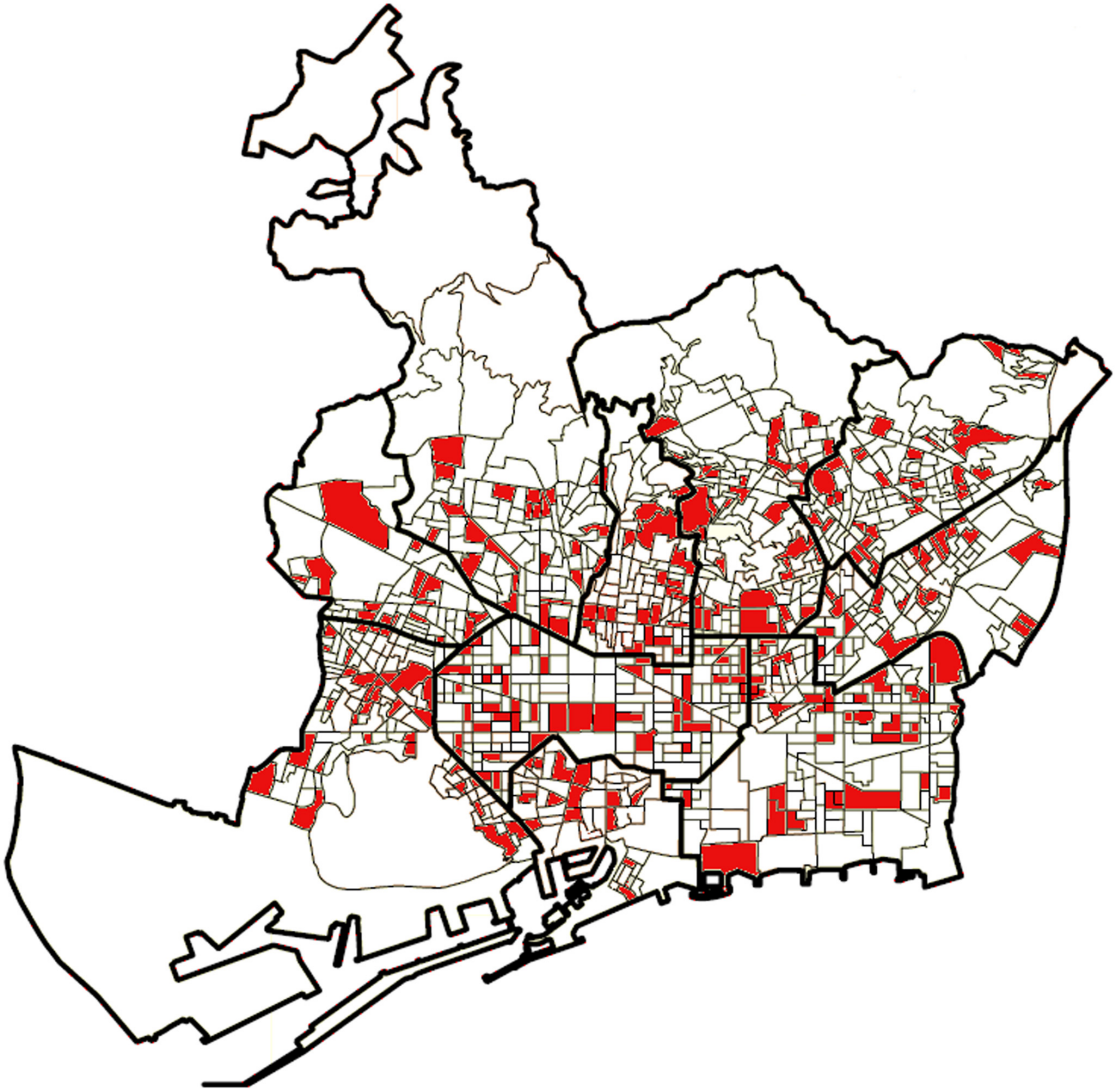
Sample of 276 electoral districts observed in the city of Barcelona (in red). Thick lines indicate administrative district borders; thin lines indicate electoral district borders.

In order to test whether a social influence mechanism is affecting the placement of flags, during July 2013 we observed the complete distribution of flags in a representative sample of 276 electoral districts (EDs henceforth) in the city of Barcelona (26% of the total); the sample includes 213,667 households in which 293,144 voters are registered. EDs are relatively small areas with a mean of 1,062 registered voters each (standard deviation = 255). The sample was stratified at the quarter level in order to ensure that all 76 quarters in the city were adequately represented. EDs were randomly selected within each quarter and the sample error is 5%, with p = q = 50% (which maximizes sample size). Six EDs were removed from the sample because they are industrial or rural areas where very few households are registered and the rest of potentially eligible EDs in the same quarters were similar. Other two similar cases were replaced randomly by other EDs in the same quarters. Fig 1 shows the extension of the sample in Barcelona’s map (see also Table 1).

**Table 1:** Aggregated sample data.

	Total	Mean per electoral district
Households^*a*^	213667	774.2
Flags	5479	19.9
2012 election^a^	Total	Mean per electoral district
Registered electors	293144	1062.1
Pro-referendum vote	123740	448.3
CIU	60504	219.2
ERC	27775	100.6
ICV	24846	90.0
CUP	8183	29.6
SI	2432	8.8
Abstention	85383	309.4

^a^ Data source: Barcelona’s City Hall statistical department.

Only Catalan national flags were registered, in their two usual versions: *senyera* (traditional version with red and yellow bars) and *estelada* (with a star). Only one flag per household was counted. Flags in commercial or office establishments, as well as in any other non-private households, were not counted. We analyzed how the density of flags correlates with the level of pro-referendum vote in the 2012 election for the Catalan Parliament, in order to see whether flag-hanging behavior tends to be inhibited in EDs where there is not a clear majority of pro-referendum vote, while it is fostered in those where such a majority clearly exists. We also analyzed whether the distribution of flag densities in the 276 electoral districts deviates from the expected distribution if pro-referendum voters display a flag with a uniform probability. Therefore, we characterize at the ED scale i) the non-linear correlation between the flag density and the density of pro-referendum voters; ii) the expected dispersion of flag densities for EDs with similar density of pro-referendum voters; and iii) the departure of the distribution of flag densities from that of an equivalent random distribution.

### Second study

We selected a sub-sample of sixteen spatial areas in different EDs. A 2×2 typology was designed and four spatial areas of each type were selected according to two criteria: density of flags (under and over the average) and type of street (wide/narrow). Most areas consist on the facades at both sides of three consecutive blocks. The resulting 82 block facades differ from each other in shape and size. The smallest number of households in a block is 4 and the larger one is 351; the average number of households per block is about 59. The data consist on a detailed daily record of the position of the flags at household resolution in the facades of the 82 blocks from 4 to 18 September 2013, followed by two additional observations, one on November 19 and the other on December 20; this makes a total of 17 different observations for each block. A total of 4,817 households were observed each of the 17 days. Note that September 11 is Catalonia’s national day (the Diada), and it is usual to hang a Catalan flag in the balcony that day. The method and criteria for counting flags were the same as in the first study. This time the records were taken on templates of the facades of the blocks, where the separation between households had been previously identified and drawn. This observation provides empirical evidence on the detailed dynamics by which flags ‘appear’ before September 11 and ‘disappear’ afterwards, departing from a given pre-existing level of flags. The intention was to measure how a global strong stimulus produces different effects on blocks subjected to different environments, in order to observe the interaction between micro-level coordination mediated by an aggregate and micro-level coordination generated at the local level (as distinguished by Schelling, 1998 [41]). Again, we analyze whether the specific spatial distribution systematically differs from the level of clustering that would be expectable by chance; we then test whether the successive hangings of flags are independent events or there is some influence mechanism that favors their clustering. So, at the balcony scale, we characterize i) the evolution of the total number of flags in the 82 observed blocks, and ii) the distribution of the clustering index (Eq 3) in the 82 observed blocks and its departure from that of an equivalent random distribution.

## Results

### Evidence of social influence at the mesoscale (electoral district level)

During the second and third weeks of July 2013 we recorded once the number of pro-referendum flags in all EDs of the sample. During this short period of time there were not any special political events able to produce noticeable changes in the number of flags, so we assume that the observations were simultaneous. These data allow to analyze the effect of social influence on the density of flags at meso-level resolution. Detailed electoral results of the 2012 election for the Catalan Parliament (see Table 1) are available for each ED. We assume that pro-referendum voters are those that in the 2012 election voted for one of the five pro-referendum parties (Convergència i Unió, CiU; Esquerra Republicana de Catalunya, ERC; Iniciativa per Catalunya Verds, ICV; Candidatura d’Unitat Popular, CUP; and Solidaritat Catalana per la Independència, SI).

**Fig 2:**
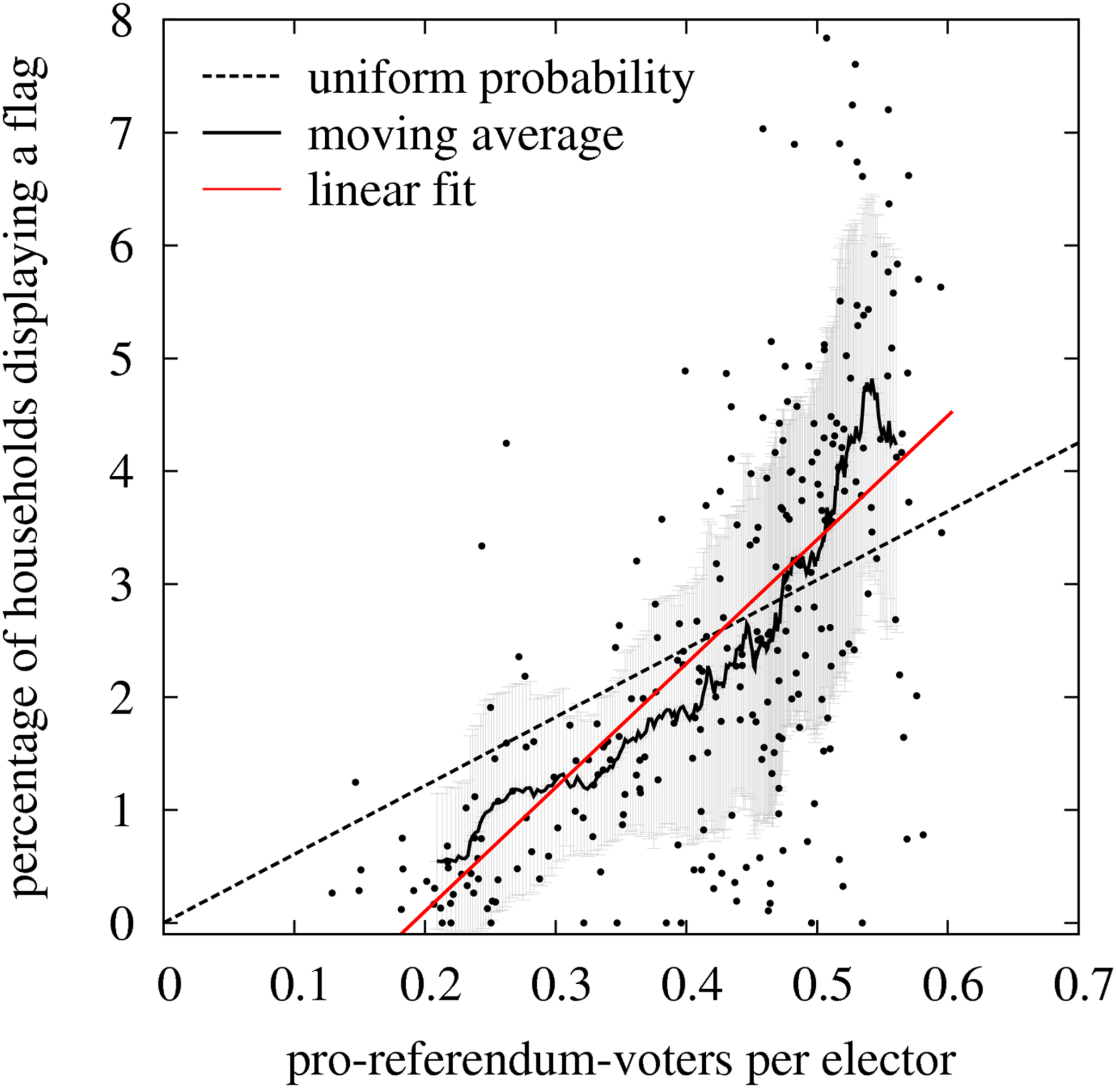
Percentage of households with a flag (100*n*_F_/*n*_H_) as function of pro-referendum voters per elector (*n*_SV_/*n*_RE_). The dots give the position of the 276 electoral districts. The dotted strike line *Y*_*u*_(*X*) = 6.075*X* represents the expected value if all pro-referendum voters have an uniform probability to hang a flag independently of the density of flags in their electoral district. The irregular curve corresponds to the moving average of subsets of 30 consecutive data points with increasing *n*_SV_/*n*_RE_ values and the gray error bars show the corresponding standard deviations of the data in each of these subsets. The red strike line is the linear fit to the 276 data points.

In the 276 observed EDs, 2.6% of households were displaying a flag when the fieldwork was done, but it must be noticed that not all households have balconies or windows at the external facade of the buildings, so if only the latter were taken into account the percentage would be higher. Note also that hanging a flag is by definition more costly that not hanging it, and that in the context of political polarization on independence it is always easier to stay anonymous; therefore it is expectable that for the majority of pro-referendum householders the default and preferred option is not signaling their political commitments. Let *n*_F_(*i*), *n*_H_(*i*), *n*_SV_(*i*), and *n*_RE_(*i*) be respectively the number of flags, households, pro-referendum votes, and registered electors in the ED *i*. The dots in Fig 2 show the relation *n*_F_(*i*)/*n*_H_(*i*) vs *n*_SV_(*i*)/*n*_RE_(*i*) for the 1 ≤ *i* ≤ 276 EDs. That is, the *Y*-axis represents the percentage of households displaying a flag whereas the *X*-axis represents the fraction of registered electors that in 2012 voted for one of the pro-referendum parties. It is evident that there is a large variation of the density of flags in EDs with similar percentages of pro-referendum voters, although in average, as expected, there is a positive correlation between *X* and *Y* (the Pearson’s correlation coefficient is *r* = 0.64). For the overall set of EDs, *N*_F_/*N*_H_ = 0,026 and *N*_SV_/*N*_RE_ = 0,422 (see Table 1) where $N_{x}=\sum_{i=1}^{276}n_{x}$. Therefore, the dashed strike line *Y*_*u*_ = 6.075*X* represents the expected percentage *Y*_*u*_ of householders displaying a flag when the fraction of pro-referendum voters is *X*, assuming that all pro-referendum householders have a uniform probability *p*_*u*_ = (*N*_F_/*N*_H_)/(*N*_SV_/*N*_RE_) = 0.06075 to hang a flag (dashed line in Fig 2), that is, an scenario without social influence. It is evident that there are less dots above the strike line *Y*_*u*_(*X*) than below, and that the absence of dots is concentrated on the left side of the plot. If the flags in the EDs were randomly distributed one would expect the dots to be evenly distributed above and below the line *Y*_*u*_(*X*). Assuming that only pro-referendum voters hang flags with a probability *N*_F_/*N*_SV_ = 0.0443, one would expect that in the plane *n*_F_ − *n*_SV_ about half of the EDs fell above the line *N*_F_ = 0.0443*N*_SV_, but only 118 of the 276 EDs do. Similarly, one would expect that in the plane *n*_F_/*n*_H_ − *n*_SV_/*n*_RE_ shown in Fig 2 about half of the EDs fell above the line 100 *n*_F_/*n*_H_ = 6.075*n*_SV_/*n*_RE_, but only 111 of the 276 EDs do. This asymmetry indicates that a local social influence mechanism is intervening in the decision of displaying a flag. The irregular growing curve in Fig 2 corresponds to the moving average *Y*_*m*_(*X*) of subsets of 30 consecutive data points with increasing *n*_SV_/*n*_RE_ values. This curve helps to visualize the non-linearity induced by social influence. At low values of *X* the EDs tend to have flag densities below the expected value *Y*_*u*_(*X*) whereas the contrary occurs at high values of *X*. An explanation for this phenomenon would be that pro-referendum voters are more likely to place a flag when they perceive they are a majority in the neighborhood. In this case, stimulation in the public expression of their political preferences would be at work in EDs where there is a clear majority of pro-referendum voters, but inhibition would be the case in EDs where there is not. Since the social influence under consideration is visually mediated through seeing flags, a more precise reasoning would be that pro-referendum voters increase their tendency to hang a flag in their balcony when they observe that in their environment there is a high density of flags, which usually, but not necessarily, occurs in EDs with high proportion of pro-referendum voters.

In order to characterize the pattern shown in Fig 2 here we define the mean relative dispersion $<\tilde{\sigma }>$ and the social influence index *I*_*SI*_. 1) The mean relative dispersion $<\tilde{\sigma }>$ = 0.58 is measured as the mean value of the ratio *σ*(*X*)/*Y*_*m*_(*X*), where *σ*(*X*) is the standard deviation of the subset of the 30 dots located around *X* and *Y*_*m*_(*X*) is the value of the moving average at *X*. 2) The social influence index is measured as *I*_*SI*_ = *p*_*lf*_/(100*p*_*u*_) − 1 = 0.81, where *p*_*lf*_ = 10.1 is the slope of the linear fit to the data (red line in Fig 2) and 100 *p*_*u*_ = 6.075 is the slope *dY*_*u*_/*dX* of the uniform probability line (black dashed line in Fig 2). These quantities are useful to characterize the observed pattern. For example, if $<\tilde{\sigma }>$ and *I*_*SI*_ are both small, the data points are all close to the uniform probability strike line *Y*_*u*_(*X*), the correlation is *r* ≃ 1, and social influence has a negligible effect on the observed variable. In our case *I*_*SI*_ > 0 which is most likely due to the fact that a mechanism of social influence is encouraging the adoption of the behavior under observation Y (in our case the density of flags in the ED) when the fraction of potential adopters *X* increases (in our case the fraction of pro-referendum voters in the ED). If instead *I*_*SI*_ < 0, it is expected that a mechanism of social influence is discouraging the adoption of the behavior *Y* as *X* increases. It is to be noticed that similar trends occur when the data are plotted in the plane *n*_F_ − *n*_SV_, but the positive curvature of the moving average is less pronounced, suggesting that what is perceived as social pressure is more the density than the absolute number of flags in the ED.

**Fig 3:**
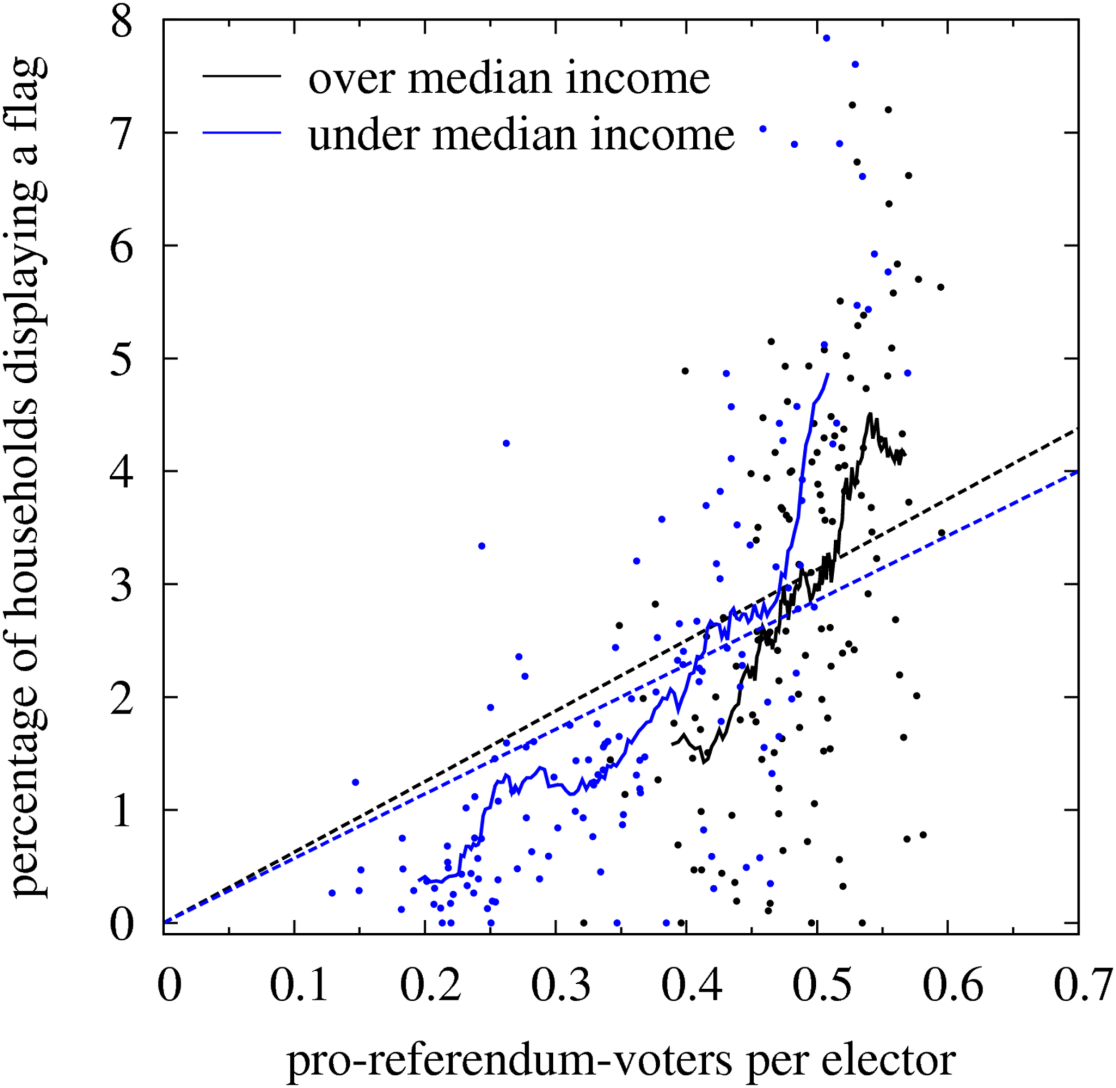
Correlation between flags pro-referendum voters for two subsets of annual income level. Income level is attributed to each electoral district from an estimation at the quarter level. The average annual income in the sample is 19,750 Eu and the median is 0.89 of this average value (source: Barcelona economia 80, November 2012, Ajuntament de Barcelona). Blue, and black symbols correspond to electoral districts with income under and over the median value, respectively. The moving average uses subsets of 20 data points instead of 30 as in Fig 2.

Fig 3 shows the same relation as Fig 2 but for two subsets of annual average household income (data taken from the Barcelona’s City Hall statistical Department; data are available for quarters, so we attribute to each ED the annual income of the quarter to which it belongs). Blue dots and curves correspond to the subset of EDs with average income below the median value, for which < *σ* > = 0.62, *I*_*SI*_ = 1.15 and r = 0.74. Black dots and curves correspond to the subset of EDs with average income below the median value, for which < *σ* > = 0.50, *I*_*SI*_ = 1.60 and *r* = 0.51. Note that the effect of local social influence manifests even more clearly in these subsets, particularly in the high income subset. This result rules out the possibility that the positive curvature of the moving average curve in Fig 2 was due to dissimilar behavioral dispositions in different income groups. Note that high-income EDs have a much narrower and higher *X* range than low-income EDs. It is out of the scope of this study to analyze the reasons and consequences of this asymmetry. Nevertheless, it is important to point out that it is not the cause of the positive value of the social influence index *I*_*SI*_ > 0 for the entire set of data. On the one hand both subsets show similar uniform probability of flag-hanging (i.e. *Y*_*u*_ = 5.72*X* for the low-income set and *Y*_*u*_ = 6.26*X* for the high-income set); on the other hand, both subsets behave similarly in the range of *X* where they overlap, even when the moving average for the high-income subset is slightly shifted downwards, suggesting that the threshold to hang a flag in average increases slightly with the household income.

**Fig 4:**
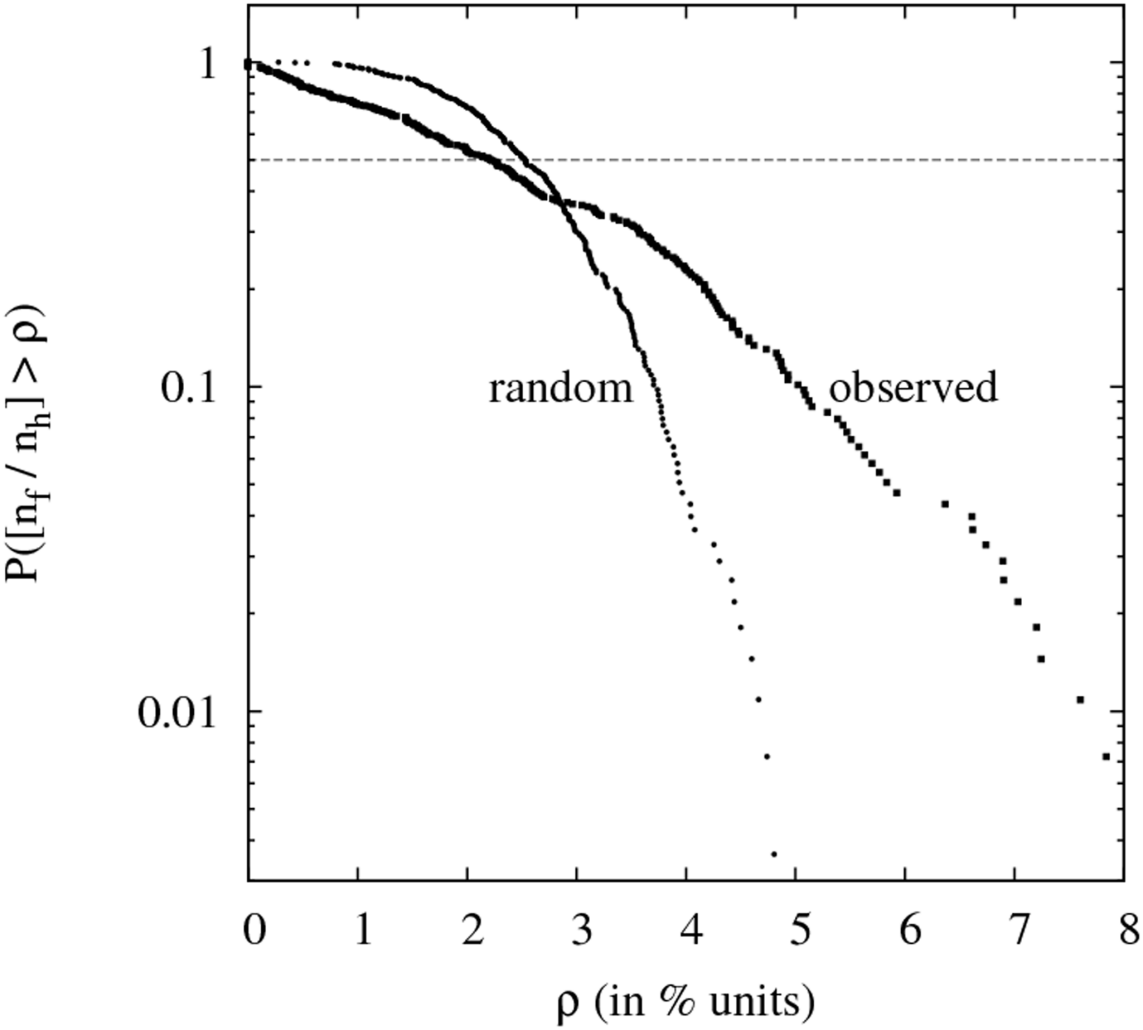
Probability $P(\frac{n_{\text{F}}}{n_{\text{H}}}>\rho )$ that the percentage of flags in an electoral district is higher than *ρ*. As indicated by the labels, the two curves correspond to the probability distribution for the observed sample of electoral districts and for a simulation where flags are placed with uniform probability *p*_*u*_ = 0.06075 (see text). The horizontal line at *Y* = 0.5 intersects the curves at their median values, 2.20% for the observed sample and 2.56% for the random sample.

Finally, in Fig 4, the distribution *P*(*n*_F_/*n*_H_ > *ρ*) represents the faction of EDs having a percentage of flags higher than a given value *ρ*. The two curves correspond to the observed distribution and to a randomly simulated one. The later is created by randomly placing flags with the uniform probability *p*_*u*_ = (*N*_F_/*N*_H_)/(*N*_SV_/*N*_RE_) = 0.06075 in each of the $n_{\text{H}}(i)\times \frac{n_{\text{SV}}(i)}{n_{\text{RE}}(i)}$ balconies of each of the 276 EDs. It is clear that the distribution *P*(> *ρ*) of the actual data deviates significantly from a random one. There is an excess of EDs with too few and too many flags. In particular, the randomly produced sample cannot explain the occurrence of 9 EDs with zero flags as observed. This deviation is in agreement with the finding presented above that low (high) density of flags inhibits (stimulates) the placement of flags, which is consistent with a process of social influence.

In this first study the unit of observation is the ED and we show that the flag distribution observed in 276 EDs cannot be explained by a linear correlation with the fraction of pro-referendum voters. On the one hand, the moving average shown in Fig 2 has a mean slope that clearly exceeds the one expected for a linear correlation with the fraction of pro-referendum voters (*I*_*SI*_ > 0). On the other hand, the dispersion of the flag densities in EDs with similar fraction of pro-referendum voters cannot be explained by the statistical fluctuations expected in a random process as demonstrated in Fig 4. All these facts strongly suggest that a mechanism of social influence is shaping the flag distribution patterns at the ED scale.

### Flag dynamics at the microscale (household level)

**Fig 5:**
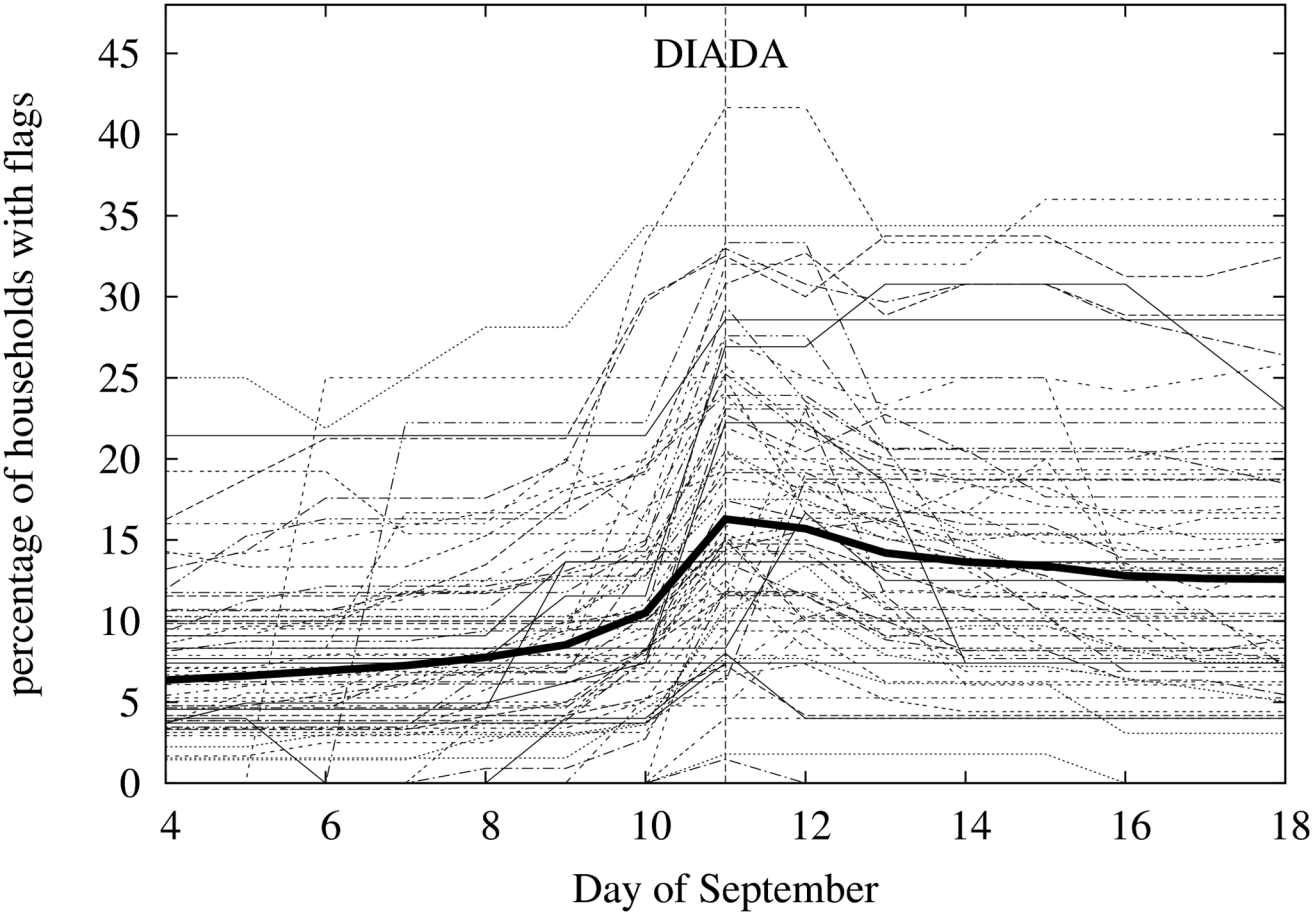
Evolution of the density of flags in a sub-sample of 82 blocks around the 2013 Diada. The thick curve corresponds to the average density of flags in all the observed households.

For our second study we observed the evolution of the detailed distribution of flags at balcony resolution in a sub-sample of 82 blocks’ facades grouped in 16 different areas. The observation took place daily from September 4 to September 18 (i.e. two weeks around the Diada) and was followed by two additional observations, one on November 19 and the other on December 20. Among the 4,817 households observed, 918 showed a flag at least once. The evolution of the number and distribution of flags in the sub-sample is strongly case dependent. Fig 5 shows the evolution of the percentage of observed households showing a flag for each of the 82 blocks. The black thick curve corresponds to the average density of flags in all the observed households. The Diada effect is reflected in a significant bump in the density of flags around September 11. However, there is a large dispersion in the density of flags among facades at any date and the evolution patterns are very diverse producing a large number of curve intersections. This case dependent behavior indicates of the complexity of the phenomena under study. Several factors may be involved in this dynamics. First of all, the initial distribution of flags on each block is different. Additionally, it is likely that potential flaggers have different participation thresholds, that is, differ each other in how much social pressure is enough for them to hang (or remove) a flag, and therefore, due to the finite size of facades, large differences in the composition of flaggers in different blocks are to be expected. Also, the finite size of facades (and other physical factors such as streets width, buildings’ height, or balconies’ visibility) may produce large differences on the particular distribution of flags on the facades. Although we expect that short range mechanisms of social influence are affecting that distribution, it is likely that the heterogeneities described above together with the global stimulus associated to the Diada tend to blur the effect of local imitation. In the following we first characterize the time evolution of the total number of flags in the sub-sample and then we find evidence of a very local effect of social influence by quantifying the departures of the clustering of flags in the observed data from that of 10000 equivalent random distributions of flags.

#### Time Evolution

**Fig 6:**
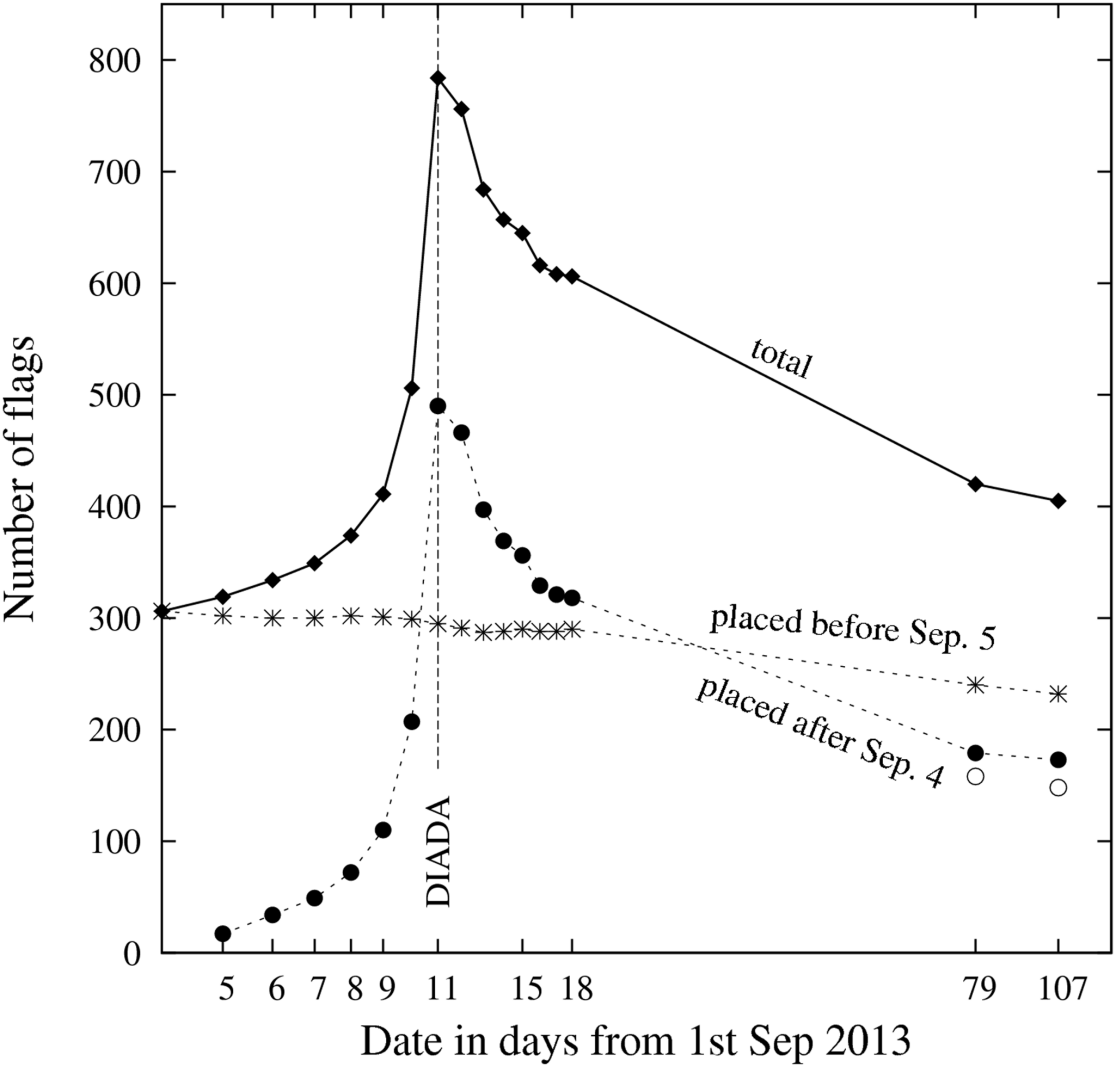
Evolution of the number of flags exposed in a sub-sample of 82 blocks. The horizontal axis is logarithmic and indicates the date of observation measured in days from September 1, 2013. Days 79 and 107 correspond respectively to the observations made on November 19 and December 20. As indicated in the labels, the square symbols represent the total number of flags, the starred symbols represent the number of flags in the households that had a flag the first day of observation, and the black circles represent the flags hanged after September 4. The two white circles below the black circle at the right end (t = 79 and 107) give the number of flags exposed on November 19 and on December 20 excluding the 21 flags that first appeared in November 19 and the eight flags that first appeared in December 20.

Fig 6 shows the evolution of the number of flags exposed in the facades of the sub-sample during the 17 observation days. The upper curve (’total’) shows this evolution in all the observed households. The two lower curves correspond to households where a flag was hanged for the first time before September 5 and after September 4: the first one, steadily decreasing, shows the evolution of the number of flags in the 306 households that had a flag the first day of observation (September 4). The second, up-and-down curve shows the evolution of the number of flags in the 612 households that hanged a flag for the first time from September 5 to December 20. During the whole observation period, 20 of the 82 blocks displayed zero flags at least one day, among which 9 never displayed any flag. Although the division of the sample into these two subsets is arbitrary, it is guided by the fact that in the previous dates to the Diada there was a strong general stimulus to support the Catalan secessionist process by displaying a flag. Within our observation period, the Diada effect seemed to induce 582 households to hang a flag. Let us call the first subset of households ‘long term flaggers’ (LTF), since they tend to keep their flag hanged for a longer period of time. Note that during the 103 days between the first and the last observation, the flags in this group decreased 100 (306–232) / 306 = 24% while for the second group the number of flags decreased 100 (582–173) / 582 = 70%, so they may be called ‘short term flaggers’ (STF), even when some (30%) may have become now LTF. The steady decreasing curve for LTF can be roughly fitted by an exponential decaying function with a characteristic time scale of about one year. That is,

$$\begin{array}{c} b_{\leq 4}\left(t\right)≃306\;e^{-(t-4)/365} \end{array}$$ (1)

where t is the time in days counted from September 1. The bump due to the Diada can be fitted as

$$\begin{array}{c} b_{>4}\left(t\right)≃\{\begin{array}{ccc} 17\; & e^{+(t-5)/1.79} & \;\;\;\;\mathrm{if}\;\;5\leq t\leq 11\\ 490\; & e^{-(t-11)/14} & \;\;\;\;\mathrm{if}\;\;11\leq t\leq 25\\ 180\; & e^{-(t-25)/365} & \;\;\;\;\mathrm{if}\;\;t>25 \end{array} \end{array}$$ (2)

where the first segment (September 5 to 11) corresponds to a rapid exponential growth with a characteristic growing time of about 2 days. The second segment (September 11 to 25) corresponds to a moderate decline with a characteristic decay time of about two weeks. The third segment represents a slow decrease after September 25 with again a characteristic declining time of about one year. The fit to this last segment is very uncertain due to the scarcity of observations in this period, but if true it would mean that among the 490 households that hanged a flag during the previous week to the Diada, about 180 became LTF. The evolution of the number of flags provides relevant information on the heterogeneous conditions under which residents in the sub-sample decided to show their support for the process. However, it should be noted that some of the 63 flags that were hanged for the first time on September 11 (the Diada) and were removed the next day are not necessarily supporting the secessionist process, since Catalans traditionally hanged a flag that day long before the process started. Likewise, the 48 flags that were removed from the balconies the same day and reappeared the next day most likely belonged to people that took these flags with them to participate in the public demonstrations of the Diada.

**Fig 7:**
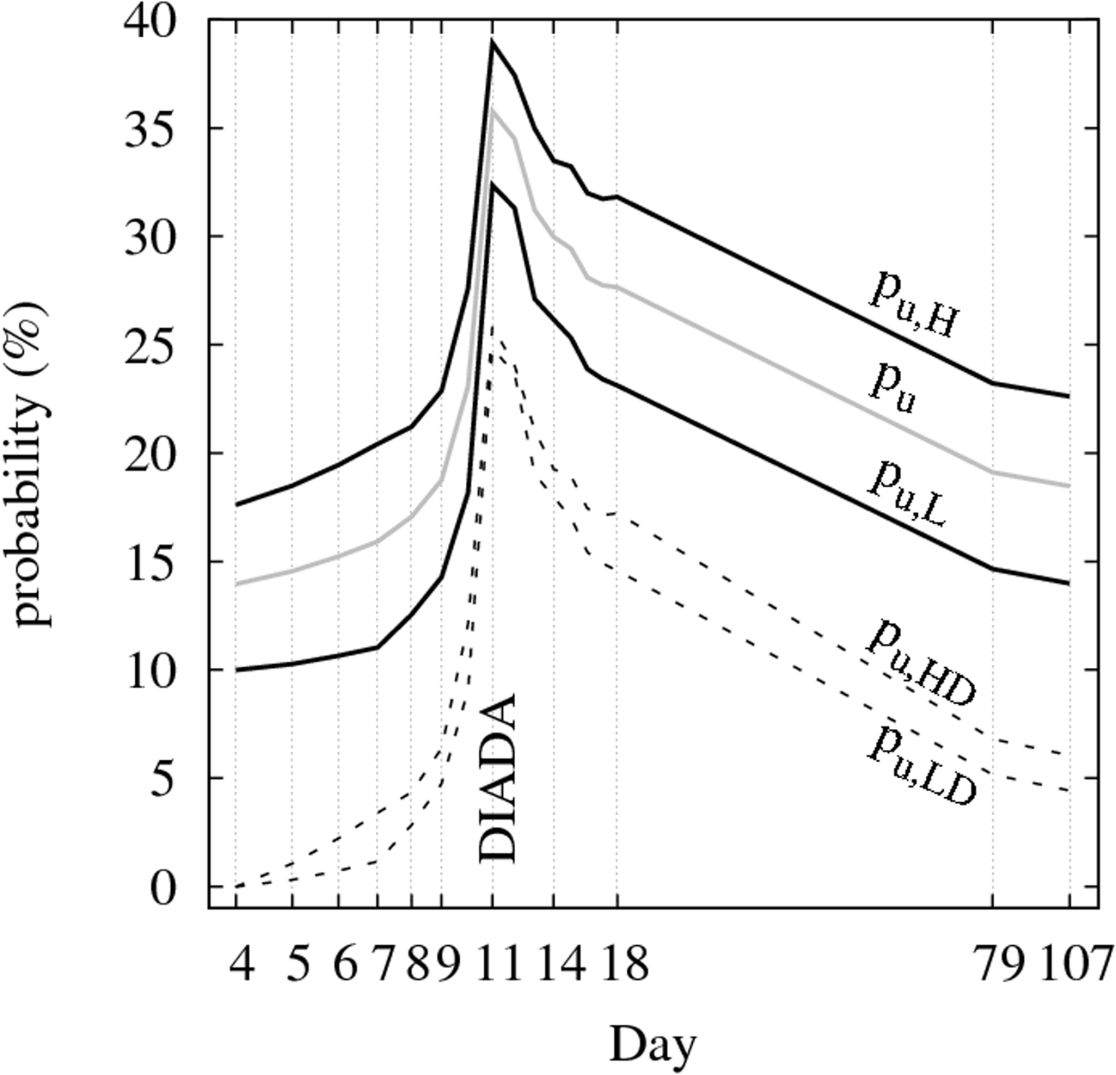
Impact of the Diada global stimulus on the uniform probability of placing flags. The gray curve labeled *p*_*u*_ shows the variation of the uniform probability in the facades of the 82 blocks due to the Diada global stimulus, where *p*_*u*_ is the probability that an average pro-referendum householder is displaying a flag at time t. The continuous curves labeled *p*_*u*, *L*_ and *p*_*u*, *H*_ show respectively the evolution of the uniform probability in the sub-samples of blocks having low (*n*_SV_/*n*_RE_ < 0.47) and high (*n*_SV_/*n*_RE_ > 0.47) proportion of pro-referendum voters. The fraction of pro-referendum voters is assumed to be the same as in the electoral districts to which the block belongs. As expected, *p*_*u*, *H*_ > *p*_*u*, *L*_ for all the dates of observation (see text). The dashed curves labeled *p*_*u*, *LD*_ and *p*_*u*, *HD*_ show respectively the evolution of the uniform probability in the two sub-samples but only for those pro-referendum householders who do not display a flag on the first day of observation.

The global effect of the Diada shown in Fig 6 can also be expressed in terms of the uniform probability *p*_*u*_(*t*), i.e. the probability that a pro-referendum householder is displaying a flag at date *t*. The gray curve in Fig 7 shows the evolution *p*_*u*_(*t*) for the entire sample of 82 blocks. During the week preceding the Diada *p*_*u*_(*t*) increased from 12.7% to 34.3%, a factor of 2.7. In the next three months *p*_*u*_(*t*) decreased to 16.9%, about half the top value at the Diada.

In order to analyse how the same stimulus (the Diada) impacts on different environments, we calculate the uniform probability in two sub-samples of blocks. To verify whether the probability that a pro-referendum householder displays a flag is enhanced in environments with high proportion of pro-referendum voters, we split the sample in two sub-samples of similar sizes, *S*_*L*_ and *S*_*H*_, the first containing the blocks with the lowest proportion of pro-referendum voters and the latter those with the highest ones. When the split is made at *n*_SV_/*n*_RE_ = 0.47 the *S*_*L*_ (*S*_*H*_) sub-sample contains 42 (40) blocks, 2589 (2228) householders and 1051 (1141) pro-referendum householders. The two continuous curves labeled *p*_*u*, *L*_ and *p*_*u*, *H*_ in Fig 7 show the evolution of the uniform probability in the *S*_*L*_ and *S*_*H*_ sub-samples, respectively. The fact that *p*_*u*, *H*_ is markedly higher than *p*_*u*, *L*_ for all dates suggests that the effects of the local influence process are not blurred by the strong global influence process. Moreover, the impact of the Diada was not exactly the same on different environments as evidenced by the different evolution of the curves labeled *p*_*u*, *LD*_ and *p*_*u*, *HD*_ in Fig 7. The probabilities *p*_*u*, *LD*_ and *p*_*u*, *HD*_ are calculated in the same way than *p*_*u*, *L*_ and *p*_*u*, *H*_ but excluding the households that displayed a flag on the first day of observation. In this way, *p*_*u*, *LD*_ and *p*_*u*, *HD*_ measure the impact of the Diada on the potential flaggers in environments with low and high presence of pro-referendum households. Interestingly, during the first 3 days of observation the growing rate of *p*_*u*, *LD*_ is almost zero whereas *p*_*u*, *HD*_ starts to grow from the first day. About two days before the Diada both probabilities start to grow quickly and reach a similar maximum value ∼ 25%. After the Diada, both probabilities start to decrease at a diminishing rate. Thus, potential flaggers in *S*_*L*_ tend to put flags later and to take them out earlier than the ones in *S*_*H*_. This behavior indicates that pro-referendum householders in the *S*_*L*_ sample are slightly less influenced by the Diada than pro-referendum householders in the *S*_*H*_. Note that as the number of flags increases, driven by the Diada stimulus, the local influence also increases, and this increase is expected to be faster in the *S*_*H*_ sample. Finally, the fact that at the Diada *p*_*u*, *LD*_(11) ≃ *p*_*u*, *LD*_(11) ∼ 25% indicates that householders in the *S*_*L*_ and *S*_*H*_ samples have similar opportunities (or limitations) to hang flags at their balconies when subjected to the same stimulus. This is a relevant fact since it helps to exclude the possibility that specific features of the buildings or the streets are causing the fostering and inhibition effects on flag-hanging in the SH and SL samples.

#### Departures from an equivalent random distribution

The results obtained for the distribution of flags at the ED level show that the decision to hang a flag is not independent of others’ decisions but subject to social influence. At the micro-scale, social influence must also be reflected in the spatial distribution of flags on the buildings’ facades. One expects the effect of social influence to be inversely proportional to the distance between the flag and the potential imitator. If this is the case, flags should have a tendency to appear together in clusters. We assume that people have a sketch of their immediate vicinity that is reinforced and updated each time they leave or return home, or stand by in front of their building; in these occasions, the presence of flags in some neighbors’ balconies is a visible fact to be remembered. On the other hand, when a potential flagger is at home and looks at the facades at the other side of the street, the flags he observes may also be relevant when deciding to hang or not a flag. It is out of the scope of this investigation to quantify the relative importance of the influence of a householder’s own facade versus that of the facades at the other side of his street. However, since posting flags is not a matter of fashion but the expression of highly polarized political preferences, it is expected that flags in the immediate vicinity will exert a larger influence than flags at the other side of the street. In any case, the influence from the observation of flags in the facades at the other side of the street is not likely to be responsible for the observed clustering of flags since the inhabitants of a block have all about the same view of the flags in the blocks in front of them.

**Fig 8:**
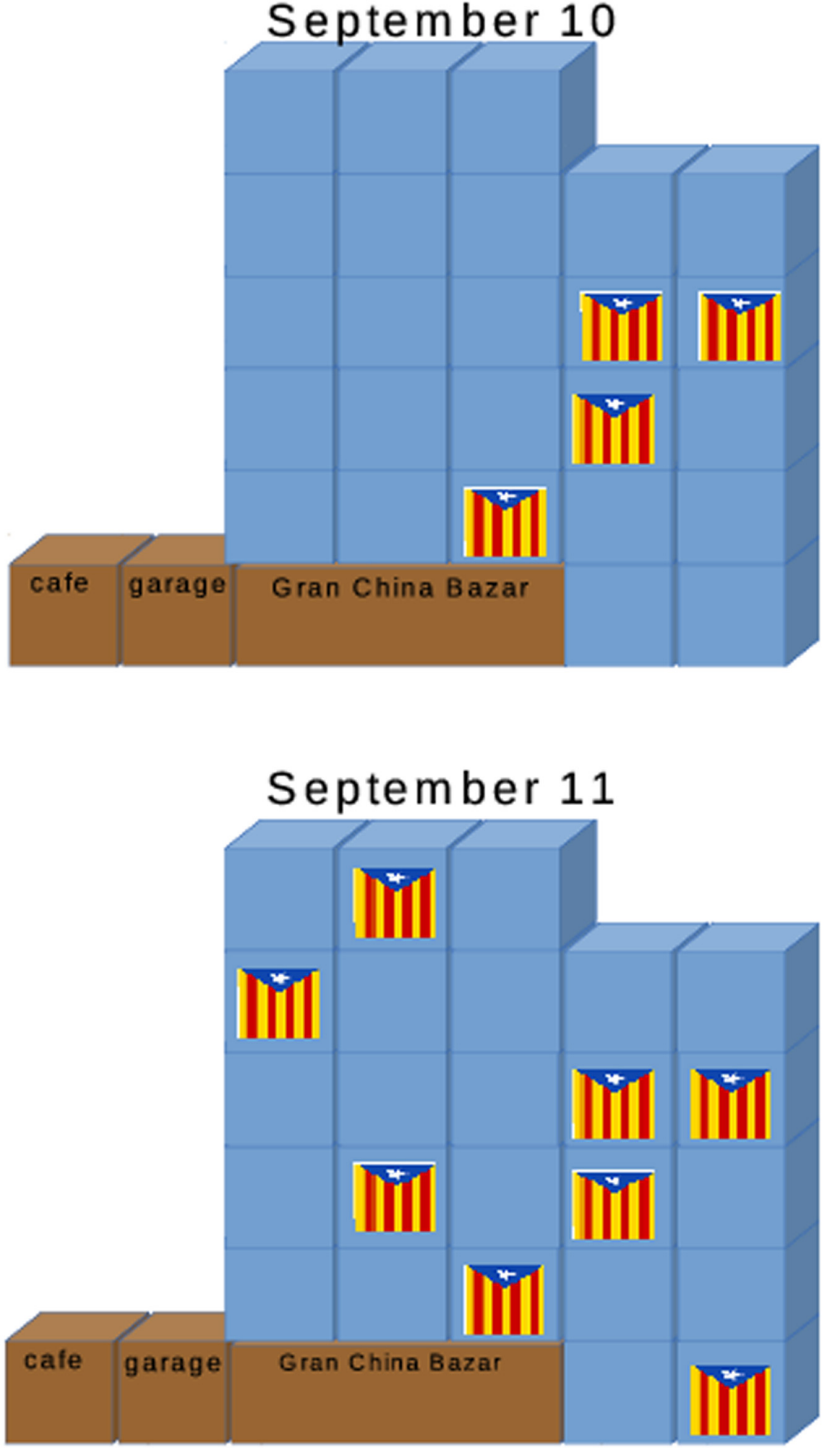
Distribution of flags on one of the smallest facades of the sub-sample in two consecutive days. We assume that the distance between horizontally or vertically adjacent balconies is one unit and therefore, as indicated in Table 2, the average of the minimal distances between flags the 10-th day is $1.104=(1+1+1+\sqrt{2})/4$ and the 11-th day is $1.362=(1+1+1+4\sqrt{2}+\sqrt{5})/8$. This is just an example that is not representative at all; there are blocks that are made of a single large rectangular building, whereas others have several buildings of different size.

There are many ways to characterize the spatial distribution of a set of points. In order to detect clustering we use the average of the minimal distances between the points *λ* (see Fig 8 and Table 2) as a simple way to estimate the tendency of flags to be close to one another; we have also tested clustering effects using pair correlation functions, but since facades differ very much in shape and size it is convenient to use a scalar measure as *λ*. Let *λ*_obs_(*k*, *t*) be the average minimal distance between the *n*(*k*, *t*) flags hanged in block *k* (where 1 ≤ *k* ≤ 82) during the date t (with *t* = September 4,5,…18, November 19 or December 20). However, *λ*_obs_(*k*, *t*) can only be defined in facades with at least two flags (i.e. cases where *n*(*k*, *t*) ≥ 2). Through all the observations (1,394: 82 blocks observed 17 times) there were 231 cases of blocks with no flags and 123 cases of blocks with only one flag. In fact, 14 blocks had less than two flags during the entire period of observation. In total, *λ*_obs_(*k*, *t*) can be calculated in 1,040 cases. Note that we are looking for a clustering tendency in a very heterogeneous set of facades. For example, a large facade with only 2 adjacent flags or a small facade full of flags both have *λ* = 1, but the probability of having 2 adjacent flags in the former case is very small whereas in the later case it is equal to 1.

**Table 2:** Clustering index for the facades in Fig 8.

Day	flags/households	*λ*_obs_	*λ*_ran_	*σ*_ran_	*C*
September 11	4/25	1.104	1.723	0.442	+1.446
September 12	8/25	1.362	1.244	0.151	−0.778

In order to circumvent this problem we homogenize the information introducing a ‘flag clustering index’ defined as

$$\begin{array}{c} C_{\mathrm{obs}}\left(k,t\right)=\frac{\lambda_{\mathrm{ran}}\left(k,t\right)-\lambda_{\mathrm{obs}}\left(k,t\right)}{\sigma_{\mathrm{ran}}\left(k,t\right)} \end{array}$$ (3)

where

$$\begin{array}{c} \lambda_{\mathrm{ran}}\left(k,t\right)=\frac{1}{10000}\sum_{i=1}^{10000}\lambda \left(k,t,i\right) \end{array}$$ (4)

and *λ*(*k*, *t*, *i*) is the value of *λ* for 1 ≤ *i* ≤ 10000 random distributions of *n*(*k*, *t*) flags in a facade with the shape of facade *k*. Therefore, *λ*_ran_(*k*, *t*) is the mean value of the average minimal distances for 10,000 random distributions and *σ*_ran_(*k*, *t*) is its standard deviation. Note that with a different meaning, the term clustering index is used for the characterization of networks and data structures.

For a given block *k* and date *t*, the clustering index *C*_obs_(*k*, *t*) measures in standard deviation units the departure of the observed distribution of the *n*(*k*, *t*) flags from the average distribution of a large number of random sets. As the density of flags on a facade increases, the average minimal distance tends to decrease, but also the standard deviation decreases. Fig 8 shows one of the smallest facades in the sample in two consecutive days. A day before the Diada there were 4 flags and the next day 4 more appeared. Table 2 shows the values of *λ*_obs_, *λ*_ran_, *σ*_ran_ and *C* for these two days.

**Fig 9:**
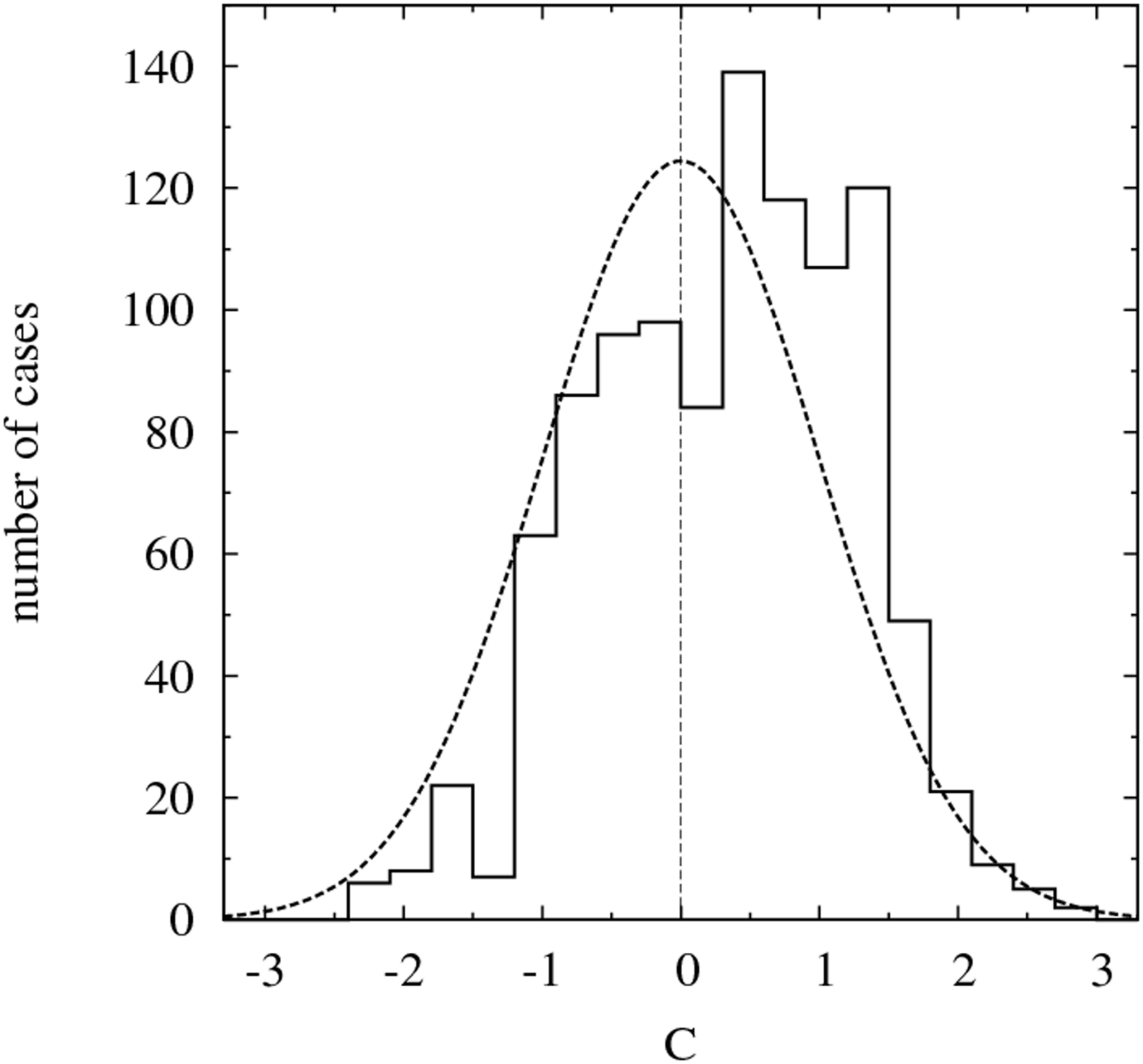
Overall distribution of the flag clustering index. The histogram represents the clustering index *C*_obs_ as defined in Eq (3) for the 1,040 observed facades. The width of bars is 0.3. The dotted curve is the normal distribution ($0.3\times 1,040\times \frac{e^{-x^{2}/2}}{\sqrt{2\pi }}$) expected for an equivalent random distribution of flags. The fact that *C*_obs_ shows a marked excess of cases with *C*_obs_ > 0 is a clear indication that globally the hangings of flags are not independent events but that a local influence mechanism is favoring their clustering.

Fig 9 shows the distribution of the 1,040 values of *C*_obs_(*k*, *t*) and the expected normal distribution for an equivalent random placement of flags as described before. We have verified that a normal distribution centered at zero with *σ* = 1 is an excellent representation of the distribution of the clustering indexes of a large set of random placements of *n*(*k*, *t*) flags on a facade with the shape of the block *k*. The clustering index for a particular random placement *i* of *n*(*k*, *t*) flags is $C_{\text{ran}}(k,t,i)=\frac{\lambda_{\text{ran}}(k,t)-\lambda_{\text{ran}}(k,t,i)}{\sigma_{\text{ran}}(k,t)}$. Note that by construction the mean value of *C*_ran_(*k*, *t*, *i*) for each block *k* and each date *t* is zero and therefore the mean value of *C*_ran_ for all the 1,040 cases is also zero. The distribution of the 1,040 values of *C*_obs_(*k*, *t*) (black histogram in Fig 9) is clearly shifted toward positive values, indicating that in overall the flags on the observed facades are markedly more clustered than expected for an average random distribution. The percentage of cases with *C* > 0 is 63%.

**Fig 10:**
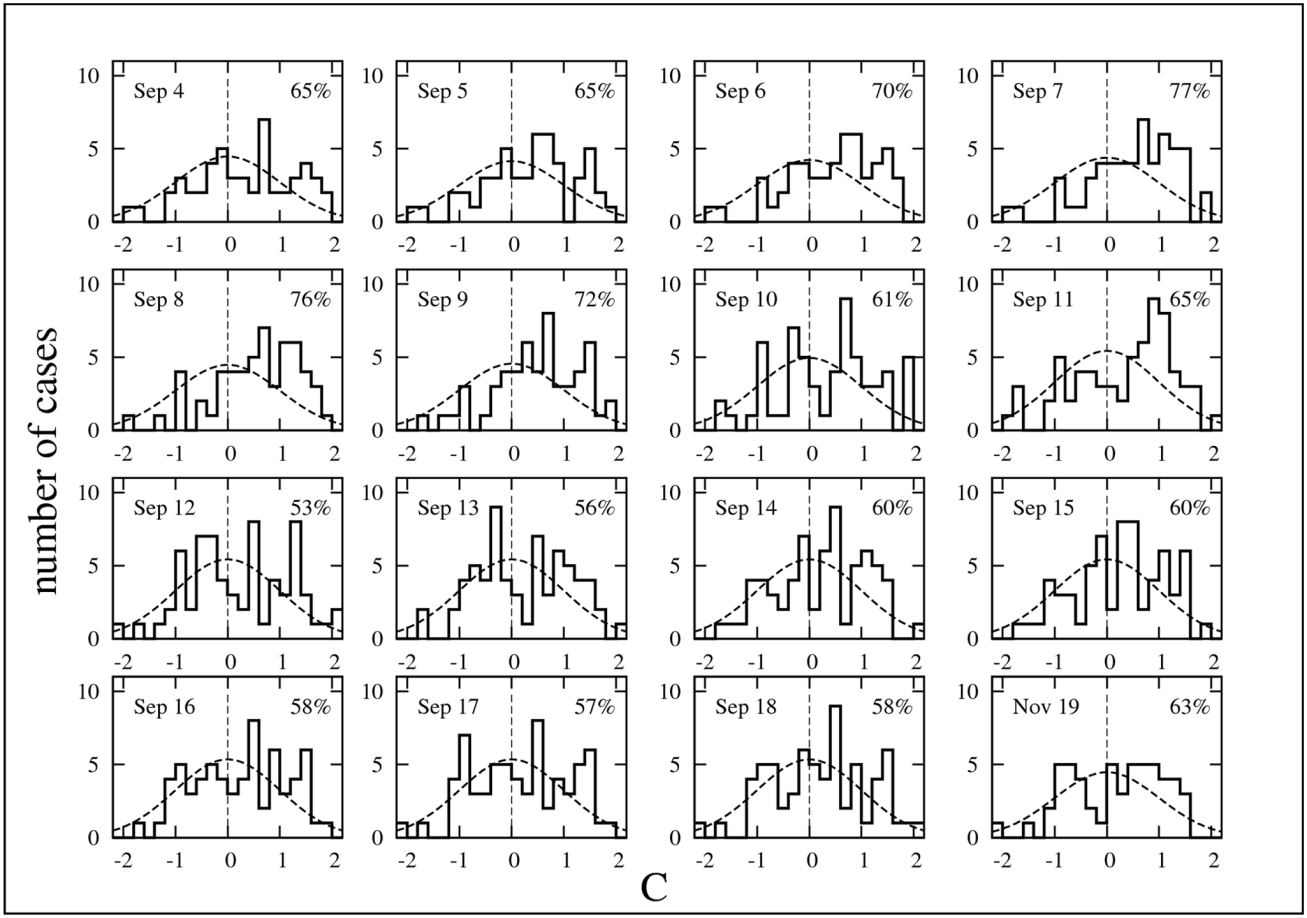
Daily distributions of the flag clustering index. Same as Fig 9 with data disaggregated by day of observation. Bin width is 0.2. The labels give the percentage of cases with *C*_obs_ > 0 and the day of the observation.

The same analysis is repeated for the observations in each single day. Fig 10 shows the frequency of occurrence of the daily values of *C*_obs_ in the blocks with at least two flags. The labels in each plot indicate the date of observation and the percentage of blocks with *C*_obs_ > 0. The results for the observations made on December 20 are not included in Fig 10 because they are very similar to the results for November 19 shown in the lower-right plot. Again, the black histograms correspond to the distribution of *C*_obs_ and the dotted curve is the normal distribution expected for an equivalent random distribution of flags for that day. All plots in Fig 10 show again a clear excess of flag distributions with *C*_obs_ > 0.

**Fig 11:**
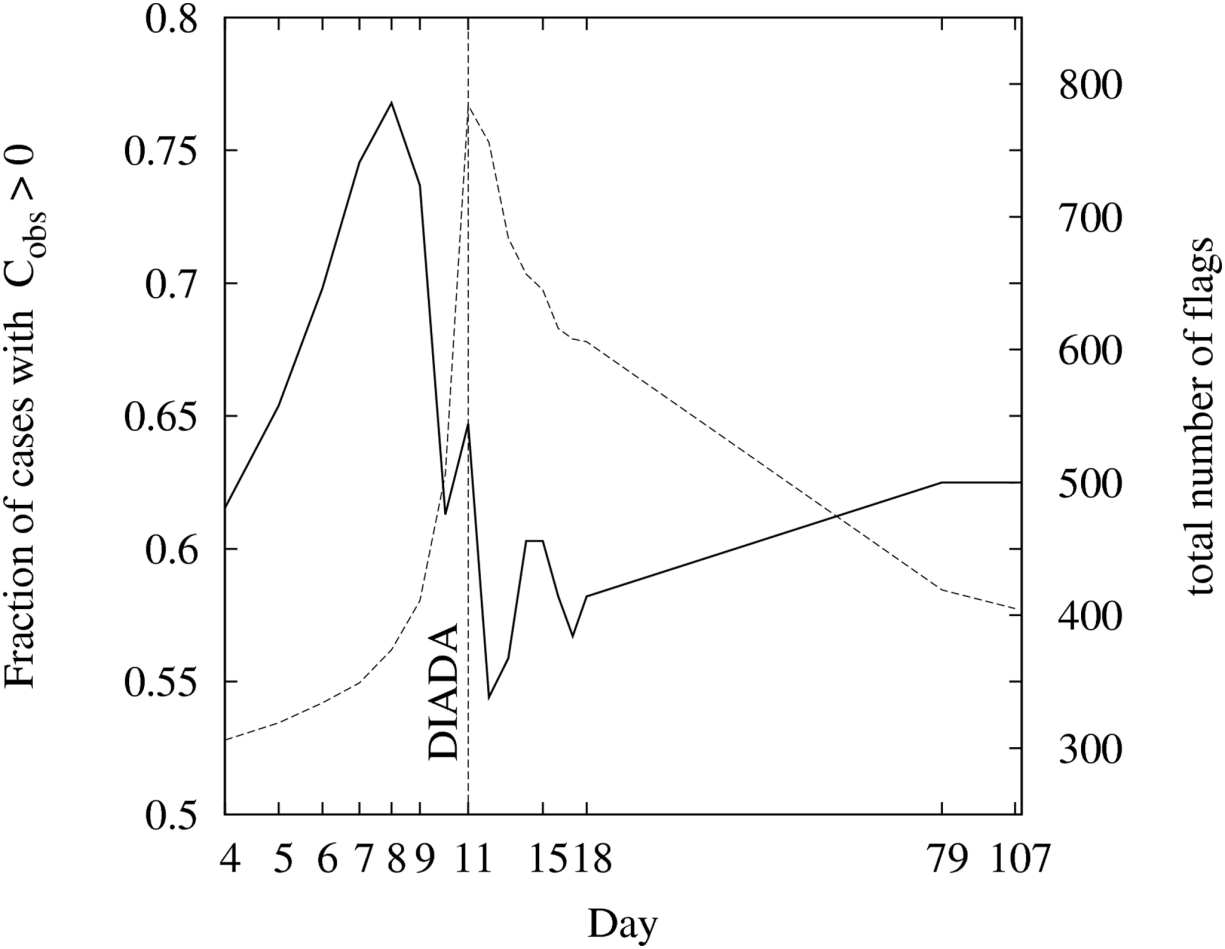
Evolution of the fraction of blocks with positive flag clustering index. The continuous curve shows the evolution of the overall fraction of cases with *C*_obs_ > 0. For comparison the dashed curve shows the evolution of the total number of flags (right-hand Y axis).

Fig 11 shows the evolution of the fraction of blocks with *C*_obs_ > 0. A strong fluctuation of the clustering is associated to a notorious increase of the number of flags due to the Diada effect. From 4 to 9 September the number of flags increases from about 300 to 400 and the clustering index increases about 10%; in the next three days the clustering index decreases about 20%, and during the following weeks it progressively recovers the values previous to the Diada.

**Fig 12:**
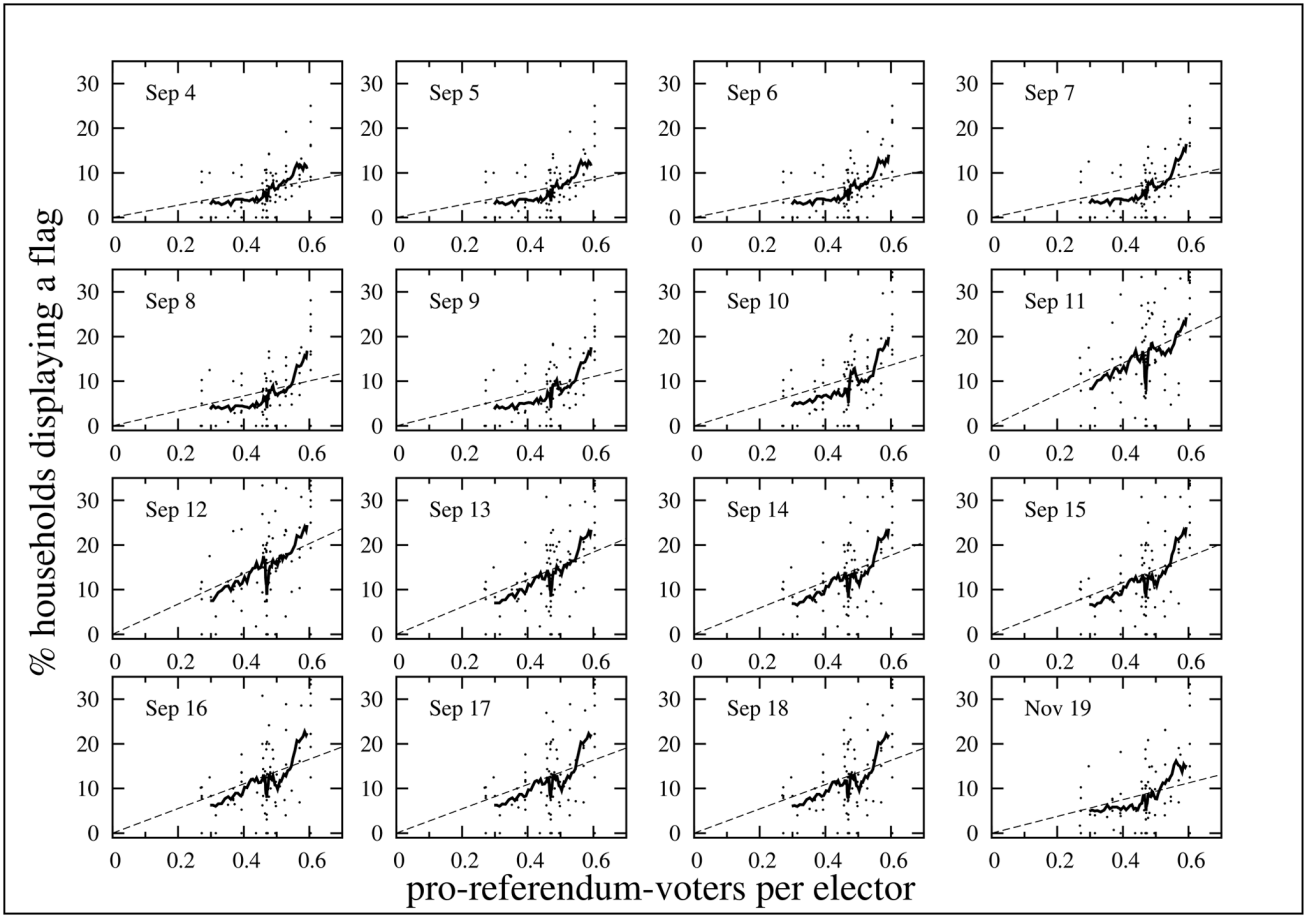
Daily percentage of households with a flag as function of pro-referendum voters per elector. Same as Fig 2 but for the flags in the facades of the 82 blocks of the second study. The labels indicate the day of observation. The fraction of pro-referendum voters is assumed to be the same as in the electoral district to which the block belongs.

Finally we have verified that the facades observed in the second study show a similar inhibition-stimulation pattern in the plane *n*_*f*_/*n*_*h*_ vs *n*_*vs*_/*n*_*ele*_ than the electoral districts observed in the first study. Fig 12 shows the percentage of observed households displaying a flag in each of the 82 blocks as a function of the fraction of pro-referendum voters. Since the information about the electoral results is available for EDs but not for facades, we assume that facades have the same electoral behavior of the EDs to which they belong. Interestingly, although the size of the sample in the second study is much smaller (82 cases, with 60 households per block in average) than the one in the first study (276 EDs, with about 770 households per ED), the observed pattern in both cases follows the same trend. The observation for the first study was performed in July 2013 during a steady political period in the sense that there was no special event that could impact on the overall number of flags in the city. Therefore, the most suitable dates of the second study to compare with the July pattern are September 4, November 19 and December 20. Comparison of Figs 12 and 2 shows that in most panels the moving average curves have a similar shape and cross the uniform probability lines in as in the first study. In fact, only the closest dates to the Diada display patterns with low indexes *I*_*SI*_. It is worth mentioning that the uniform probability *p*_*u*_ in both studies cannot be directly compared since on the one hand the number of flags in the city was not the same, and on the other hand the number of households in the first study includes all households in the ED, whereas in the second study we only count those with a balcony/window in the facade.

The results of this second study show that the effect of social influence is affecting both the distribution of flags in a facade (excess of blocks with positive clustering index) and the block distribution pattern in the plane *n*_*f*_/*n*_*h*_ vs *n*_*vs*_/*n*_*ele*_. It is likely that the same micro-mechanism is responsible for these two effects even when the first manifests at the micro-scale whereas the second manifests at the meso-scale. Additionally, the fact that the observed number of cases with zero flags (9 EDs in the first study and 20 facades in the second study) largely exceeds the number expected by random indicates that there is a strong inhibitory social pressure against being the first to publicly manifest your political preference in your neighborhood.

## Discussion

We have documented and characterized a process of social influence in the expression of political preferences departing from direct observation in the field. Two observational studies on how people hang pro-referendum flags in Barcelona in the context of the Catalan secessionist process provide data to support the claim that the probability that a private household’s balcony or window shows a flag when the inhabitants have secessionist preferences is significantly affected by how many neighbors at the local level have hung a flag. The first study at the ED level shows that there is an inhibition-stimulation dynamics in flag-hanging, depending on whether pro-referendum voters are or not majority in each ED. Departing from the correlation between flag density and vote for pro-referendum parties, flags appear more frequently than expected in districts with a majority of pro-referendums voters, and less frequently than expected in districts where such a majority does not exist (in fact, the excess of EDs with zero flags is also indicating that a social inhibition effect is operating in the latter districts). Since a social influence mechanism at the ED scale is likely to be visually mediated, a more precise reasoning would be that pro-referendum voters have an enhanced tendency to hang a flag when they observe that in their environment there is a high density of flags, which usually, but not necessarily, occurs in EDs with a high proportion of pro-referendum voters. This is in line with Nowak et al (1990) [42] dynamic social impact theory, which predicts that an individual will be more likely to conform to the preferences and behavioral propensities of the local numerical majority, and that this produces the clustering of attitudes and behaviors.

The second study at the balcony-window level allows to isolate short term flaggers from long time flaggers and to show that social influence is also reflected in how flags are distributed on the buildings’ facades. We calculated a clustering index in order to show that flags tend to appear more clustered than expected by chance. The clustering index adopts positive values when the average minimal distance between flags in a facade is smaller than the value expected by random, and we find that there is a clear excess of facades having positive values. This indicates a very local social influence effect that reaches its maximum when the potential flagger sees a flag placed at his side. This excess of positive clustering index values persists for all the observation days showing that the successive hangings of flags are not independent events but that a local influence mechanism is favoring their clustering. It is somewhat surprising that the effect of local social influence manifests so clearly in this second study as well as in the first one (see Fig 2), when certainly other non-local mechanisms of social influence may play a role in the decision of displaying a flag, such as mass media information, political discussion with acquaintances living in other EDs, or the observation of the flag density in other areas of the city. It is out of the scope of this work to quantify the relative importance of local, non local and global processes of social influence, but the results described above indicate that local social influence is important in determining the social attitudes that individuals adopt in their environment. Note also that a local social influence effect at the level of blocks or buildings is not necessarily a social contagion effect as defined in the introduction, since it does not have to be based on a dyadic interaction. However, this is not to deny that a dyadic social contagion mechanism may be responsible for the posting of some flags, particularly for those individuals who have very low thresholds for expressing their political preferences. Arguably, both mechanisms, together with other global stimuli such as media effects, may be acting simultaneously on individuals’ decisions to hang flags.

Alternative explanations usually confounded with social influence [43] such as homophily could hardly explain the observed patterns. At the ED scale a possible effect of homophily in terms of income level is discarded since the inhibition-stimulation dynamics persists when controlled by this variable (see Fig 3). At the block scale a possible effect of clustering produced by the segregation of the potential flaggers in terms of their thresholds to hang a flag is highly unlikely. On one hand, people residence’s decisions were taken generally long time ago, and on the other hand, the information about neighbors’ tendency to publicly express their political preferences is not a relevant factor when making that decision (not to mention it is not accssesible). The possibility of differentiated contextual effects can also be ruled out since the Catalan process and the related political struggles and discussions to which all residents in the city are exposed are essentially the same; there are not specific mass media at the level of EDs or neighborhoods, so specific exposure to mass media sources within or without pro-secessionist EDs is not plausible: pro-secessionist voters should be expected to follow their preferred mass media wherever they live. Besides, there are not systematic or obvious differences between the features of the buildings and streets in pro-secessionist EDs compared with the rest. Although the data at hand cannot tell us exactly what the source of the observed non-linear pattern is, we cannot think of a simpler and more plausible explanation than social influence.

A limitation of the studies presented is that they do not allow inferring the specific type of social influence mechanism in operation. This is a usual shortcoming in social influence studies, since different mechanisms are typically compatible with observed patterns of influence [19]. However, the two studies presented here provide detailed quantitative information that can be used to constraint the functional form of the mechanisms of social influence that drive the flag dynamics. The more suitable strategy to test different mechanisms is through agent-based simulations, since they allow to produce simulated patterns that can be compared to the observed data through characterizing quantities such as the clustering index C, the social influence index *I*_*SI*_ and the correlation coefficient r, as well as the local and global evolution of the number of flags. However, although there may be different social and psychological mechanisms operating, they all lead to a similar behavioral rule: do A if enough members of the relevant group do so. We have provided evidence that this behavioral rule is operating in our case.
